# Primary intraosseous squamous cell carcinoma of the maxilla possibly arising from an infected residual cyst: A case report

**DOI:** 10.3892/ol.2014.2644

**Published:** 2014-10-27

**Authors:** SHINTARO SUKEGAWA, HIDENOBU MATSUZAKI, NAOKI KATASE, TAKAHIRO KANNO, TOSHIKO MANDAI, YUKA TAKAHASHI, JUN-ICHI ASAUMI, YOSHIHIKO FURUKI

**Affiliations:** 1 Division of Oral and Maxillofacial Surgery, Kagawa Prefectural Central Hospital, Takamatsu, Kagawa 760-8557, Japan; 2 Department of Oral Diagnosis and Dentomaxillofacial Radiology, Okayama University Hospital, Okayama 700-8558, Japan; 3 Department of Molecular and Developmental Biology, Kawasaki Medical School, Kurashiki, Okayama 701-0192, Japan; 4 Department of Oral and Maxillofacial Surgery, Shimane University Faculty of Medicine, Izumo, Shimane 693-8501, Japan

**Keywords:** primary intraosseous squamous cell carcinoma, residual cyst, computed tomography

## Abstract

Primary intraosseous squamous cell carcinoma (PIOSCC) is a rare type of odontogenic carcinoma arising from the jawbone. Odontogenic cysts are true cysts that arise from the dental epithelium, which is associated with tooth formation. The epithelial lining of odontogenic cysts has the potential to transform into various types of odontogenic tumor; however, this transformation from an odontogenic cyst to a malignant tumor is rare. The definitive diagnosis for PIOSCC generally requires the observation of either features of squamous cell carcinoma (SCC) within the jawbone that are distinct from direct invasion from the surface oral epithelium, or evidence of SCC arising from odontogenic epithelium and from tumors that have metastasized to the jawbone from distant sites. In the present study, a case of PIOSCC of the maxilla is presented, which, based on the results of computed tomography and the clinical course, was hypothesized to have originated from an infected residual cyst.

## Introduction

A primary intraosseous odontogenic carcinoma, which is a term that was recommended by the World Health Organization (WHO) in 1972 (1) is a type of squamous cell carcinoma (SCC) arising within the jawbone that purportedly develops from remnants of odontogenic epithelium. In 2005, the WHO classified these lesions as odontogenic carcinomas, termed primary intraosseous SCC (PIOSCC), and divided them into three types: solid type; keratocystic odontogenic cyst-derived; and odontogenic cyst-derived (2). A definitive diagnosis of PIOSCC is difficult as the lesion must be distinguished from tumors that have metastasized to the jawbone from distant sites, from alveolar carcinomas that have invaded the bone from the surface and from tumors of maxillary origin (3,4).

Odontogenic cysts are true cysts that arise from the dental epithelium, which is associated with tooth formation. The epithelial lining of odontogenic cysts has the potential to transform into various types of odontogenic tumor (5,6). However, transformation from an odontogenic cyst to a malignant tumor is rare (5,6).

The current study presents a case of PIOSCC of the maxilla that, based on the results of computed tomography (CT) and the clinical course, was hypothesized to originate from an infected residual cyst. Written informed consent was obtained from the patient.

## Case report

In September 2006, a 45-year-old male underwent extraction of the upper left, first and second premolars at a dental clinic (Takamatsu, Japan). In March 2012, the patient identified a gingival swelling in the upper left premolar region and was referred to another general dental practitioner (Takamatsu, Japan). At that clinic, the patient underwent incision and drainage of the lesion following clinical diagnosis of a dental infection. However, there was no improvement following the treatment and thus repeat curettage of the lesion was performed. Following these treatments, the lesion continued to grow gradually. In July 2012, the patient was referred to Kagawa Prefectural Central Hospital (Takamatsu, Japan) with swelling and mild pain in the upper maxilla. The patient had no medical or surgical history. The patient smoked 20 cigarettes a day and has consumed two alcoholic beverages per week for the past 15 years. The patient’s family history was noncontributory.

**Figure 1:**
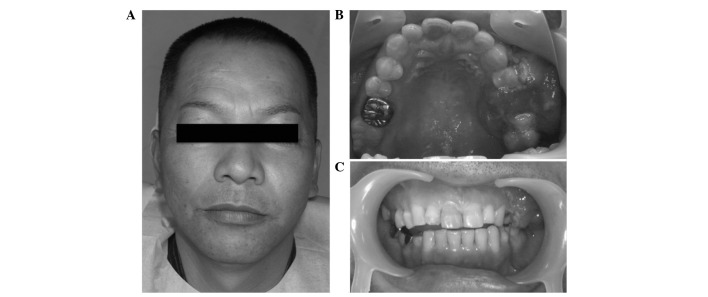
(A) Marginal facial asymmetry was observed on the left side of the patient’s face. (B and C) Intraoral images were captured showing a large mass located in the buccal and palatal aspect of the edentulous alveolus of the left maxilla, in the area between the second premolar and the first molar. The mucosal surface was covered with rough hemorrhagic papules, which were pink-red in color.

Extraoral examination revealed marginal left-sided facial asymmetry and tenderness (Fig. 1A); however, the patient experienced no abnormal sensation in the left buccal area. The left submandibular and upper jugular lymph nodes were palpable and tender. The intraoral examination revealed a mass, 25×35-mm in diameter, located in the buccal and palatal aspect of the edentulous alveolus of the left maxilla, in the area between the second premolar and the first molar (Fig. 1B and C). The mucosal surface of the mass was rough and covered with small and protruding hemorrhagic papules, which were pink-red in color. On palpation, the mucosa surrounding the mass appeared to be normal and was not indurated. However, tenderness and bleeding were identified.

**Figure 2:**
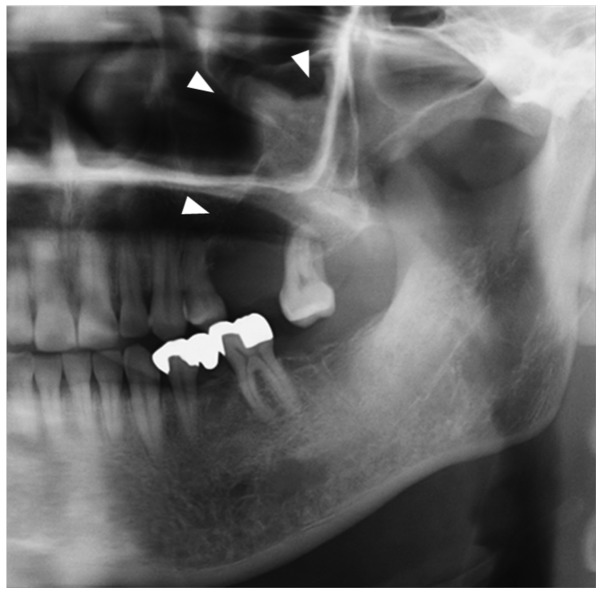
A panoramic radiograph revealed a dome-shaped radiopaque mass with well-defined margins extending from the left maxilla to the maxillary sinus.

**Figure 3:**
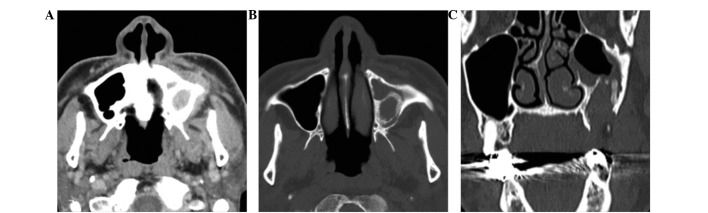
Computed tomography images. (A) Axial soft tissue window images revealed a cystic lesion extending from the left maxillary alveolar area to the maxillary sinus. (B and C) Axial and coronal bone window images revealed the thickened floor of the antrum, which was elevated. (C) A section of the elevated sinus floor was destroyed.

**Figure 4:**
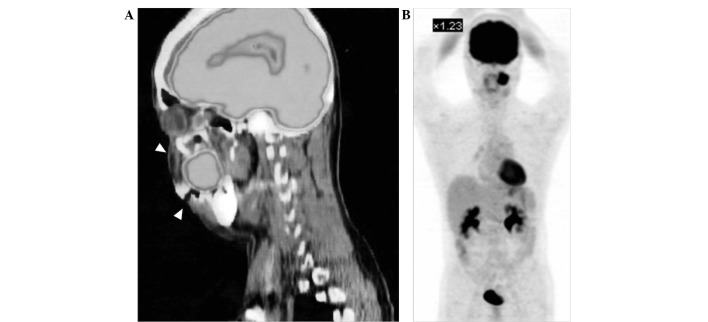
(A) Axial 18^F^-fluorodeoxyglucose positron emission tomography (FDG-PET)/computed tomography revealed FDG accumulation in the lesion in the left maxilla (maximum standardized uptake value, 12.2). (B) No other abnormal FDG accumulation was detected elsewhere by FDG-PET.

A panoramic radiograph revealed a dome-shaped radiopaque mass with well-defined margins extending from the left maxilla to the maxillary sinus. The lesion caused the floor of the antrum to be elevated (Fig. 2). CT revealed a round cystic lesion, 30×40 mm in dimateter, which extended from the left maxillary alveolar region to the maxillary sinus (Fig. 3A and B). The floor of antrum was elevated by the cystic lesion and its margins were thickened (Fig. 3B and C). A section of the elevated sinus floor had been destroyed (Fig. 3C). 18^F^-fluorodeoxyglucose-positron emission tomography (FDG-PET) detected FDG uptake in the left maxilla [maximum standardized uptake value, (SUV_max_), 12.4; Fig. 4A] and in two submandibular lymph nodes (SUV_max_, 2.2). No abnormal FDG uptake that would have been indicative of another primary tumor or distant metastasis was detected on the FDG-PET images (Fig. 4B).

From these imaging results, the lesion was diagnosed as a primary malignant tumor arising from the left maxilla. The tumor was clinically staged as T4aN2bM0 in accordance with the 2009 Union for International Cancer Control system (7). An incisional biopsy was performed and indicated that the lesion was a PIOSSC. The patient underwent a subtotal maxillectomy of the left maxilla, and left radical neck dissection under general anesthesia and the diagnosis was histopathologically determined following surgery.

**Figure 5:**
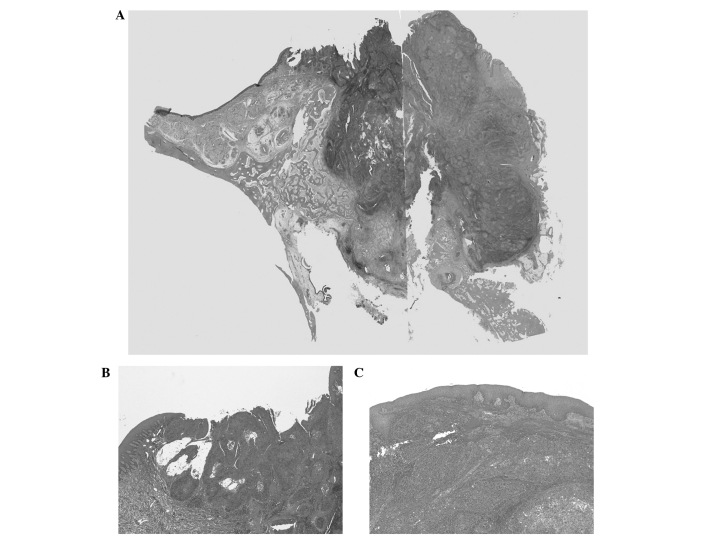
Histopathological observations. (A) The tumor mass was located in the center of the maxilla and extended to the surface epithelium. The epithelium of the maxillary sinus was not involved. (B) The tumor cells formed atypical squamous epithelium, exhibiting features of squamous cell carcinoma (magnification, ×2). (C) The surface of the mass was covered by non-cancerous oral mucosa with ulcers, indicating an intraosseous origin (magnification, ×2).

The histopathological examination of the excised specimen revealed tumor cells consisting of atypical squamous epithelial cells with enlarged nuclei, which had invaded the submucosal connective tissue and bone (Fig. 5A–C). The tumor was accompanied by necrosis inside the tumor nests and scattered mitotic figures (Fig. 5D and E). These features indicated a poorly differentiated SCC. Inside the lesion, there was no clear evidence of the presence of cysts. However, the tumor had progressed to the surface from deep within the tissue, no atypical cells were observed in the epithelium at the boundary of the ulcer, and there was no contiguity between the oral mucosal epithelium and the maxillary sinus mucosa. No histological metastasis to the lymph nodes was identified. Based on these findings, the lesion was finally diagnosed as PIOSCC.

## Discussion

PIOSCC is a rare odontogenic tumor of the jawbone arising from residual odontogenic epithelium, initially without connection to the oral mucosa. In 2005, the WHO (2) categorized PIOSCC into three types: solid type; keratocystic odontogenic tumor-derived; and odontogenic cyst-derived.

Gardner (8) and Hampl and Harrigan (9) reported that the most important criterion for the diagnosis of primary intraosseous odontogenic carcinoma is the presence of a transition zone between the normal and malignant epithelia. In the present case, a transitional area between the normal oral squamous epithelium and the SCC was observed and, histopathologically, the tumor cells were not contiguous with the oral mucosal epithelium or the maxillary sinus mucosa. As a diagnostic criterion for primary intraosseous odontogenic carcinoma, previous studies have proposed the exclusion of other primary tumors (3,4). FDG-PET is considered to be a useful modality for evaluating malignant tumors, as well as the primary site, lymph nodes and occurrence of distant metastases. In the present case, marked FDG uptake (SUV_max_, 12.4), indicating that the tumor was malignant, was detected only in the maxilla and no other abnormally high uptake was observed elsewhere. Therefore, the lesion was diagnosed as a PIOSCC within the jawbone.

The incidence of PIOSCC derived from odontogenic lesions is complicated to determine. If the disease is not in its early stages, it is difficult to demonstrate the actual site of malignant transformation. At later stages, the carcinoma may destroy the structures of the original lesion (8). In the present case, the existence of odontogenic epithelium was not revealed histologically. CT demonstrated elevation of the floor of the antrum, which revealed destruction that was caused by the lesion. Considering the CT findings and the clinical course, it was hypothesized that the carcinoma had developed from an odontogenic cyst or benign tumor in the maxilla. Therefore, this case was hypothesized to be a PIOSCC derived from an odontogenic cyst or keratocystic odontogenic tumor.

The pathogenesis of PIOSCC remains unclear. It has been hypothesized that the key factor in carcinogenesis is chronic inflammation from the infection of odontogenic lesions (8,10,11). Infection and inflammation may contribute to carcinogenesis via three major factors: i) Formation of reactive oxygen and nitrogen species by phagocytes that subsequently damage DNA, proteins and cell membranes; ii) infectious agents may directly transform cells by inserting oncogenes into the host genome, inhibiting tumor suppressor genes or stimulating mitosis; and iii) infectious agents may induce immunosuppression and thereby reduce immunosurveillance (12,13). PIOSCCs and oral mucosal carcinomas express a different set of oncogenes and tumor markers, indicating different genetic pathways (14). In the present case, inflammatory cell (lymphocyte and neutrophil) infiltration was identified within the stromal components, which may have been caused by the incision and drainage of the lesion that had been performed previously. However, these pathological findings did not indicate the existence of chronic inflammation. CT revealed bone thickening of the elevated floor of the maxillary sinus and these bone changes indicated chronic inflammation. In addition to the abovementioned CT observations, the findings of the present case indicated the past existence of a lesion that had been infected for a long period. Therefore, we hypothesized that the PIOSCC was derived from a radicular (residual) cyst of an inflammatory cyst, which had the potential for infection.

In the updated WHO (2005) classification (2) odontogenic carcinomas are generally divided into four categories: ameloblastic carcinoma; PIOSCC; clear cell odontogenic carcinoma; and ghost cell carcinoma. Of these, ameloblastic carcinoma is classified as primary- or secondary-type ameloblastic carcinoma. The primary type of ameloblastic carcinoma arises *de novo*. The secondary type, malignant transformation of ameloblastic carcinoma, is considered to occur from a recurrent or pre-existing ameloblastoma. Karakida *et al* (15) proposed that chronic inflammation following surgical treatment may lead to a malignant transformation, resulting in the secondary type of ameloblastic carcinoma. In the current case, it was hypothesized that chronic inflammation caused the cyst-lining epithelium to undergo malignant transformation. Therefore, the present case may have occurred secondary to a residual cyst.

In conclusion, the current study presents a case of PIOSCC of the maxilla, which, based on the CT findings and its clinical course, was potentially derived from a residual cyst. Clinicians must be aware that odontogenic cysts that are subject to chronic inflammation have the potential to undergo malignant transformation.
